# Location, location & size: defects close to surfaces dominate fatigue crack initiation

**DOI:** 10.1038/srep45239

**Published:** 2017-03-27

**Authors:** Itziar Serrano-Munoz, Jean-Yves Buffiere, Rajmund Mokso, Catherine Verdu, Yves Nadot

**Affiliations:** 1 Université de Lyon, INSA-Lyon, MATEIS CNRS UMR 5510, Villeurbanne Cedex, 69621, France; 2 Paul Scherrer Institute, CH-5235 Villigen-PSI, Switzerland; 3 Institut Pprime, CNRS, ISAE-ENSMA, Université de Poitiers, Département Physique et Mécanique des Matériaux, Téléport 2, 1 Avenue Clément Ader, BP 40109, F-86961 Futuroscope Chasseneuil Cedex, France

## Abstract

Metallic cast components inevitably contain defects such as shrinkage cavities which are inherent to the solidification process. Those defects are known to significantly alter the fatigue life of components. Yet very little is known, quantitatively, on the dangerosity of internal casting defects compared to surface ones. In this study, fatigue specimens containing controlled internal defects (shrinkage pores) are used to foster internal cracking. *In situ* fatigue tests monitored by X ray synchrotron tomography revealed that the internal nucleation and propagation of cracks was systematically overran by surface cracking initiated at castings defects up to ten times smaller than the internal ones. These findings indicate that the presence of internal defects in cast components can be tolerated to a larger extent than is allowed by nowadays standards

Casting processes are extensively used by the transportation industry for the production of components with complex shapes, such as internal passageways, otherwise costly to machine in a part made form a wrought product. Al-Si cast alloys are particularly attractive because of their versatility (the amount of Si can be adjusted to obtain different properties) and low cost to weight ratio. However, the use of cast metals in structural components is hampered by the presence of casting defects (*i.e.,* pores and/or oxides)^1^,^2^,^3^,^4^,^5^,^6^,^7^,^8^. These defects are inherited from the solidification process and their presence in the final products is detrimental to the material mechanical resistance, especially in the case of cyclic loading (fatigue) during service life. Producers of cast components have established standardised non-destructive procedures to discard those components containing the most dangerous defects based on a comparison with standardised X ray radiographs (as, for example, the ASTM E155-15 standard^9^). The rejection criteria is based on the shape and size of defects regardless of their location: in contact with the ambient air or buried deep into the material bulk.

The fatigue life of any material can be divided into the nucleation of (at least) one crack, its propagation and the final failure^10^. At low stress levels, the main fraction of the lifetime is spent in the nucleation period which is a defect-controlled process. Defects act as local stress raisers fostering crack nucleation and, depending on the size of the defect, the nucleation period can be even suppressed. Therefore, the total fatigue life of cast aluminium alloys can be considerably reduced due to the presence of casting defects.

The propagation period depends on the intensity of the driving force (induced by the remote stress field) at the crack-tip, as well as on the surrounding environment. It has been observed since long (first studies date back to 1917) that gases have a deleterious effect on fatigue life. In fact, for Al alloys, crack growth rates are the lowest when cycling is performed in vacuum and increase when the environmental conditions are changed, for example, to dry air or ambient air^11^,^12^,^13^,^14^,^15^,^16^. Crystallographic and serrated fracture surfaces are characteristic of vacuum conditions while ambient air conditions produce rough and flat surfaces^17^.

For the case of cast Al alloys, literature shows that fatigue cracks are principally nucleated at surface and/or subsurface casting defects^18^,^19^,^20^,^21^,^22^,^23^. When no such defects are present, crack nucleation is induced by other microstructural parameters such as favourable grain orientations or Si particles^24^,^25^. Internal failure in cast Al alloys is however rarely observed during high cycle fatigue (10^5^ < N_*cycles failure*_ < 10^7^) and, when it occurs, it does not entails a reduction of the expected fatigue life^26^,^27^. As for the environmental conditions of internal cracking, they are generally considered to be close to those of vacuum as their fracture surface features look similar^26^,^28^,^29^.

Further knowledge on the initiation and propagation of internal fatigue cracks is crucial to safely promote the use of cast solutions in critical structural components. Also, more accurate criteria during non-destructive controls will promote a reduction of the rejection percentages. In this work we aim to measure internal crack growth rates by using fatigue specimens which contain controlled internal casting defects. X ray tomography is essential to tackle the problem. First, it is necessary for manufacturing the fatigue specimens (laboratory tomography), and more importantly, it enables the monitoring of internal crack nucleation and propagation (synchrotron X ray Phase Contrast Tomography (PCT))^30^,^31^,^32^,^33^,^34^,^35^. Computerized Tomography (CT) scans of the consecutive propagation stages are used to measure (and compare) internal and surface crack growth rates in the same experimental conditions and the differences between internal and surface cracking are discussed.

## Results

As explained in the Methods section, the fabrication of suitable fatigue specimens is a complex and time consuming process. All specimens that have been tested are summarized in Table 1. The internal defects contained in these specimens are microshrinkages whose size is measured through the square root of the defect surface (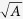)^36^ projected along the loading axis.

**Figure 1:**
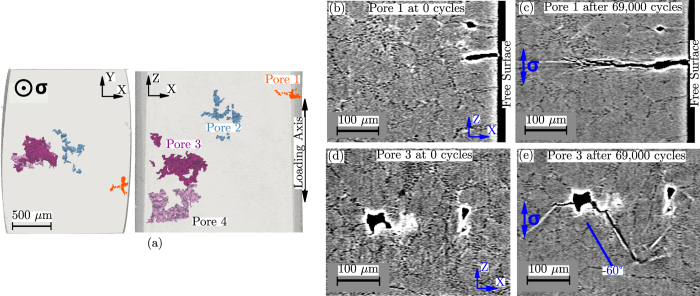
(**a**) 3D rendering of the pores within the gauge of SLS-14 specimen. (**b**) 2D *μ*CT reconstructed slices showing Pore 1 state at 0 cycles and (**c**) after 69,000 cycles. (**d**) Pore 3 state at 0 cycles and (**e**) after 69,000 cycles. The dark microscopic patterns (apart from the pores and cracks) visible on the images correspond to the eutectic Si particles. The white contour (and some straight lines) around pores and cracks is due to phase contrast.

None of the five specimens presented in Table 1 failed from the propagation of an internal crack although two of them did initiate and propagate some internal cracks. Of all five specimens, SLS-14 specimen produced the most interesting internal propagation events. The pore distribution within its gauge is shown in Fig. 1a. Pore 1 corresponds to a surface defect of 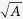 = 120 *μ*m which nucleated a crack (hereafter named Crack 1) after 40,000 cycles. Pore 2 (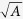 = 210 *μ*m) nucleated a small internal crack (~15 *μ*m) after 63,000 cycles when the crack tip of Crack 1 was approaching. It is thought that this nucleation is due to the influence of the crack-tip plastic zone. Pore 3 (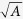 = 340 *μ*m) nucleated an internal crack (Crack 3) after 40,000 cycles. Finally, Pore 4 (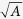 = 300 *μ*m) did not nucleate any crack.

The differences in crack path morphology between internal and surface propagation are shown in Fig. 1b–e. The crack path resulting from surface propagation at Pore 1 is relatively rough and the growth direction is almost perpendicular to the loading axis (Z). On the contrary, the internal crack which nucleated from Pore 3 exhibits large facets and oblique growth (in this case at −60°): a crack morphology similar to what is obtained under vacuum conditions^26^. It must be noted that, occasionally, some perpendicular growth can be also observed around Pore 3 (Fig. 1e, left crack) although internal propagation has been observed to be predominantly faceted-like.

**Figure 2:**
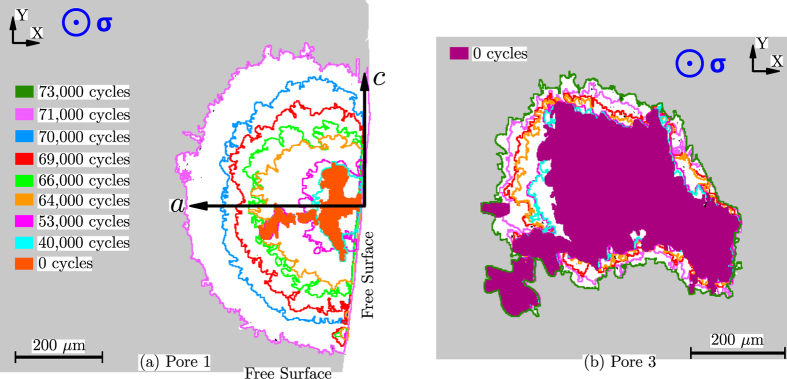
(**a**) Propagation stages of the surface crack nucleated at Pore 1 (projected views). For simplicity, the last propagation stage of Crack 1 at 73,000 cycles is not shown because the crack continued propagation throughout the bottom edge. (**b**) Propagation stages of the internal crack nucleated at Pore 3.

Figure 2 shows the propagation stages of Crack 1 and Crack 3. The crack front of Crack 1 has a typical elliptical shape with a *a*/*c* ratio of 1.1. On the other hand, the shape of Crack 3 front is much more irregular. The nucleation is not uniform around the perimeter of Pore 3. During the first stages (from 40,000 to 64,000 cycles), several crack fronts are created at the top, left and bottom of Pore 3. After 70,000 cycles the isolated crack fronts meet and form a unique crack front that spans along most of the pore perimeter.

**Figure 3:**
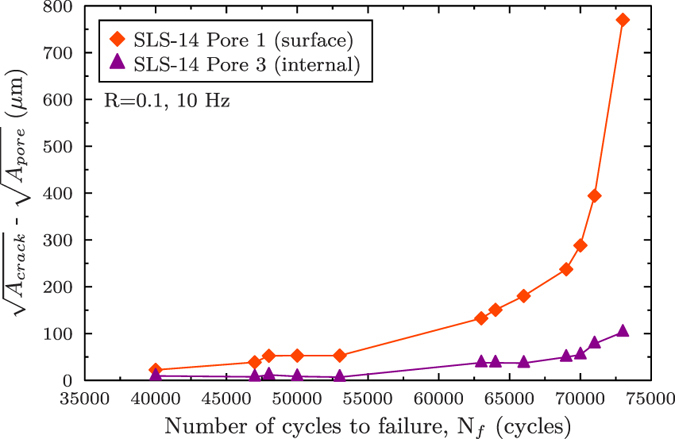
Plot of the evolution of the square root of the projected surface of the cracks (the square root projected surface of pores is subtracted) against the number of cycles to failure for Pore 1 and Pore 3.

The crack length is measured by taking the square root of the crack surface projected along the loading axis. For comparison purposes, the square root of the projected surfaces of Pore 1 and Pore 3 (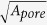) are subtracted. The results are shown in Fig. 3, where it can be observed that the internal crack growth is slower than the surface growth. For the first 53,000 cycles, the differences in growth are not very large but after 64,000 cycles, Crack 1 grows much faster than Crack 3. At 73,000 cycles, Crack 1 is almost eight times larger than Crack 3. The crack growth rate has also been evaluated using linear measurements of the crack length *a* performed every 20° around the two crack fronts: the average of the linear measurements *a* (over 10 counts for Crack 1 and 16 for Crack 3) are in good agreement with the results shown on Fig. 3 and show the same trend, *i.e.,* a slower crack growth rate for the internal crack^37^.

**Figure 4:**
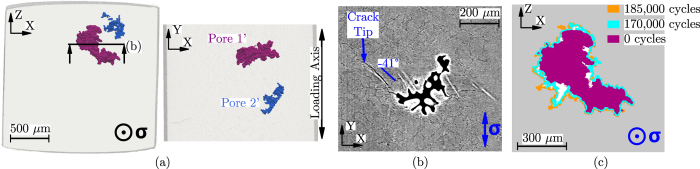
SLS-17 specimen: (**a**) 3D rendering of the pores within the gauge. (**b**) 2D *μ*CT reconstructed slice showing Pore 1′ state after 185,000 cycles. (**c**) Propagation stages of the internal crack nucleated at Pore 1′.

The SLS-17 specimen was the other specimen where some internal propagation was observed. Figure 4a shows the specimen gauge where two main pores are present. Only Pore 1′ (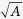 = 386 *μ*m, the 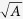 of Pore 2′ is 230 *μ*m) nucleated an internal crack (Fig. 4b) which propagated at 41° and was only observed after 170,000 cycles. Figure 4c shows a crack front even more irregular than the one observed in Fig. 2b. It seems that in SLS-17 internal propagation is favoured in a small region around Pore 1′ perimeter, which might indicate that the local crystallographic orientation can also influence internal crack growth, although the local crystallography surrounding the pore was not investigated in this work.

## Discussion

The method to prepare samples containing controlled natural defects, albeit tedious and with a high rejection rate, turned out to be successful for obtaining fatigue specimens containing internal defects. Before testing, one would have considered these defects ideal to foster internal crack initiation and propagation (see for example specimen SLS-22 of Fig. 7). Nevertheless, as explained in the previous section, it has hardly been possible to observe internal crack initiation (and propagation) from those specimens, for the experimental conditions investigated in this work. Moreover, final failure was never caused by the internal defects probably for two main reasons. First, internal crack nucleation is retarded. An internal pore three times larger than the surface one is required to induce simultaneous crack nucleation (at 40,000 cycles for SLS-14). Secondly, surface crack growth rates are higher (see Fig. 3) than those measured on internal cracks.

**Figure 5:**
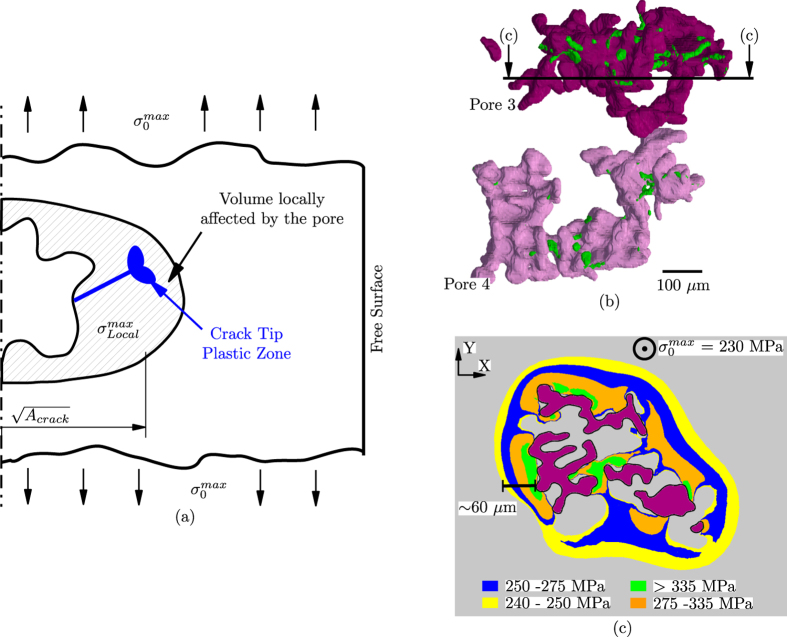
(**a**) Schematic illustration of an internal pore which nucleated a crack that remains engulfed within the pore mechanically affected region. 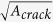 is the projected area of the internal pore and crack, 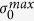 is the maximal remote stress and 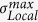 is the maximal local stress within the locally affected region. (**b**) 3D rendering of FE simulation on SLS-14 Pore 3 and Pore 4 showing (in green) the yielded volume with stresses higher than 335 MPa (for the shake of clarity, only these high values are shown). (**c**) FE simulation of SLS-14 Pore 3 showing the distribution of local *σ*_zz_ stress values above the nominal stress level in the region located approximately at the crack initiation site.

A thorough discussion of all parameters influencing the crack nucleation period (*e.g.,* pore size and shape, local crystallography) is beyond the scope of this article. Nevertheless, Finite Element (FE) simulations based on the real 3D pore geometry (see ref. 37 for details on the meshing process) were performed to investigate the mechanical differences between the four pores of SLS-14 specimen. The yielded volume is the region where the local stress exceeds the yield stress of the material (*σ*_*YV*_ > 275 MPa, not to be mistaken with the volume mechanically affected by the pore as explained in Fig. 5a and c) is used to compare the ability of the four pores to nucleate a crack based on the activation of plastic deformation. Figure 5b shows, in green, this yielded volume around Pore 3 and Pore 4. It results that the yielded volumes of SLS-14 Pore 3 (internal, one crack, 9,391,494 *μ*m^3^) and SLS-14 Pore 4 (internal, no crack, 1,792,151 *μ*m^3^) are respectively 10 and 2 times larger that that of SLS-14 Pore 1 (surface, one crack, 903,560 *μ*m^3^). The yielded volume of SLS-14 Pore 2 (internal, no crack, 423,276 *μ*m^3^) is the smallest. Therefore, these results suggest that, after 73,000 cycles at *σ*_*max*_ = 230 MPa (R = 0.1), an internal pore needs to produce an apparently larger plastic zone size (at least ten times) to be able to nucleate a crack at the same time than a surface pore. Smaller yielded volumes (those of Pore 2 and Pore 4) do not lead to internal crack nucleation and it is unknown if these pores would be able to produce any nucleation at higher numbers of cycles.

Regarding the internal defects, it has been shown above that the pore which has nucleated the crack is the one with the largest yielded volume. So that if the size of the yielded volume is a good criterion to rank the internal pores with respect to crack nucleation, this criterion obviously does not “work” when considering internal and surface pores. The reason why a defect like Pore 1 nucleates a crack in spite of a smaller yielded volume has probably to do with the presence of the free surface where a larger slip irreversibility is expected because of the air environment. The effect of air/vacuum is, of course, not accounted for in the FE calculations.

**Figure 6:**
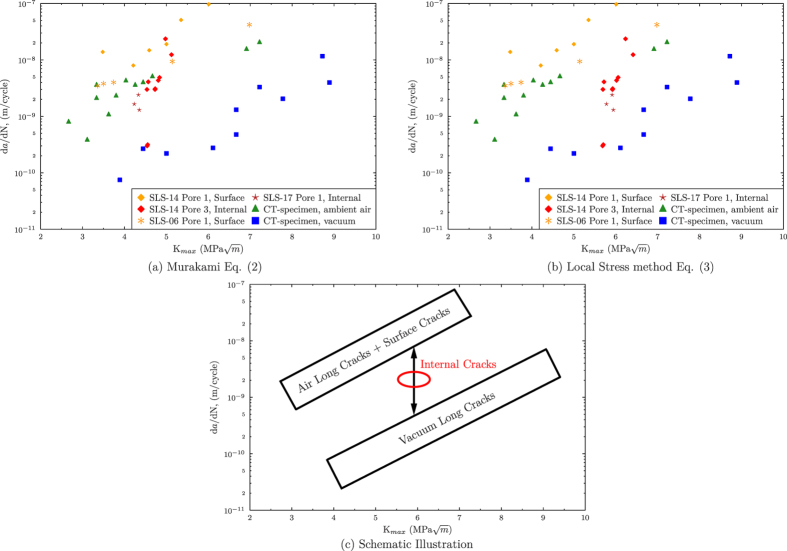
Plot of the crack growth rates against the maximum stress intensity factors for: (**a**) The K_*Imax*_ of internal cracks calculated using the Murakami empirical equation (Eq. 2 shown in red). (**b**) The K_*Imax*_ of internal cracks calculated using the Local Stress method (Equation 3, shown in red). (**c**) Schematic illustration of the three main tendencies observed depending on the environment and the position of the pore.

Compact Tension (CT) specimens of the same alloy were used to obtain the reference d*a*/dN against K_*max*_ curves for both ambient air and vacuum (secondary vacuum p ≈ 5 × 10^−4^ Pa, see Fig. 6 with triangle and square symbols respectively).

The stress intensity factors K_*max*_ of the surface cracks monitored during *in situ* testing at the synchrotron are calculated using the following empirical correlation between *K*_*Imax*_ and 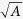 proposed by Murakami^38^:


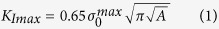


where 0.65 is the geometry correction factor calculated for inclined surface cracks of arbitrary shape, 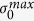 is the maximal remote applied stress and 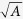 is the crack surface. These calculation results are shown in Fig. 6a,b (with orange diamonds and asterisks) and it can be observed that they superimpose quite well with the data obtained with the ambient air CT-specimen.

Internal propagation rates are first calculated using the following empirical equation, also proposed by Murakami^38^:


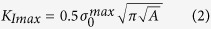


where 0.5 is the geometry correction factor for an arbitrarily shaped 3D internal crack propagating in mode I. The results of this calculation are shown in Fig. 6a, in red. These results are puzzling as they tend to indicate that, for equivalent K_*Imax*_ values, the internal cracks propagate with crack growth rates similar to those of surface cracks. However, many literature results^11^,^13^,^14^,^17^,^26^,^27^,^28^,^29^,^37^, as well as our CT-specimen results, indicate that internal cracks which are assumed to propagate in a vacuum like environment, grow with rates about one order of magnitude lower than surface cracks (ambient air propagation).

It is likely, however, that the small cracks observed in Figs 2b and 4c remain engulfed in the pore local stress field 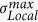 during all the experiment (see Fig. 5a). Hence, the following equation (referred as the Local Stress method) is suggested in order to be able to calculate the driving force of these cracks:


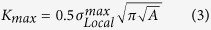


Figure 5c shows a slice of SLS-14 Pore 3 where the intersections of the pore with the plane of the figure are shown in magenta. The values of the volume mechanically affected by the pore (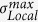 > 240 MPa) are shown in yellow (ranging form 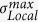 = 240 MPa to 250 MPa), blue (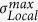 = 250–275 MPa), orange (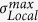 = 275–335 MPa) and green (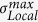 > 335 MPa). The grey colour corresponds to stress values between 230 and 240 MPa. The average values of 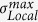 are calculated for both SLS-14 Pore 3 and SLS-17 Pore 1. The simulation results show that the average of the maximal local values for SLS-14 is 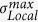 = 288 MPa (where the remote nominal stress value is 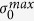 = 230 MPa) and 273 MPa (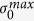 = 200 MPa) for SLS-17. These values are obtained by calculating the mean of all the nodes surrounding the pore for which 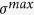 values are higher than 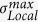 > 240 MPa (or 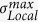 > 210 MPa for SLS-17). Also, we choose to use the resultant *σ*_*zz*_ component in Eq. 3, rather than the Von Mises stress, because the driving force at the crack tip is mostly influenced by the crack opening displacement. The differences between *σ*_*zz*_ and 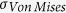 calculations are not very large, although the affected region (*i.e.,* the volume around the pores where the local stress values are higher than the remote applied stress 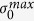) obtained by 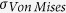 is slightly smaller.

The stress intensity factors calculated using the Local Stress method are shown with red diamonds and stars in Fig. 6b. One can see from this figure that the propagation curves of the internal cracks move forwards the vacuum CT data when the local stress values are taken into account, although they do not superimpose with vacuum data. Figure 6c illustrates this shift towards higher K_*max*_ values. In other words, it seems that, for the same diving force, the internal crack growth rates are higher than the vacuum long cracks ones, but remain slower than those of surface cracks.

Although the environmental conditions of internal cracks are not precisely known, it is certain that they influence the internal crack growth rates. It has been observed^29^ for titanium alloys, where internal crack nucleation is induced by the microstructure itself and not by defects, that internal propagation exhibits slower crack growth rates than surface propagation occurring under the highest vacuum level possibly attained using *state-of-the-art* pumps. This might indicate that for titanium alloys the internal vacuum is purer than that produced at the pumped free surface.

Because of their convoluted morphology, the internal defects studied in this work (Figs 1 and 4) are considered to be micro-voids (therefore vacuum-like environment) occurring from a volume deficit during solidification. On the contrary, gas pores are relatively spherical *bubbles* which result from a reduced solubility of hydrogen with decreasing temperatures in the melt. Somehow it is assumed that these two types of pores are fundamentally different^39^. Yet, the reality is more complicated and mixed pores can form and grow under the combined action of shrinkage and gas. Thus, SLS-14 Pore 3 could be a mixed pore containing certain amount of hydrogen that, once the crack nucleated, will increase the internal growth rate until it is all absorbed by the crack tip. The presence of hydrogen within the pores could explain why internal crack growth rates are lower than in air but higher than in primary vacuum (see Fig. 6c).

The material of study was chosen mainly because of the important amount of literature on the influence of surface/subsurface defects, and also because aluminium alloys allow to work with intermediate X ray energies (~25 keV) which are available in many synchrotron facilities around Europe. The results presented above (dominance of surface defects) can be applied to any material containing defects of any kind (*e.g.,* pores, oxides or inclusions). However, it is of special interest for cast alloys because this fabrication process favours the occurrence of internal defects over surface ones. As well, the reader must be aware that the important difference in internal/surface behaviour observed is this article can be reduced if the surface environment changes to more inert conditions. This is the case of, for example, aircraft components that will spend most of their fatigue life in dry air conditions^13^. In this case, the harmfulness of internal pores probably needs to be reassessed.

All in all, this study brings up some good news for casting producers. We have shown that, because of the retarded crack nucleation and growth rates induced by the environment, internal defects do not reduce the expected fatigue life the way surface defects do when cycling in ambient air conditions. Moreover, it seems that one of the conditions for internal failure to occur is that the surface must be free of any surface/subsurface casting defect.

## Methods

### Material

The alloy of study is an A357-T6 cast aluminium provided by CTIF^40^ in the form of rods of *ϕ*30 × 250 mm. The chemical composition (wt%) is: Balance Al, 6.94 Si, 0.56 Mg, 0.097 Fe, 0.13 Ti, <0.015 Cu, <0.03 Mn and <0.003 Pb. The heat treatment after casting consisted in: (a) solution treatment at 540 °C for 10 h in an air circulated furnace; (b) water quenching at room temperature and; (c) artificially ageing to the peak age condition at 160 °C during 8 h. Regarding the microstructural parameters: the Secondary Dendrite Arm Spacing (SDAS) is 38 *μ*m, the average grain size is *ϕ*_*eq*_ = 500 *μ*m (area fraction measurement), and the pore volume fraction is 0.002%. As for the mechanical parameters: the ultimate tensile strength is *σ*_*UTS*_ = 335 MPa, the yield strength *σ*_*y*_ = 275 MPa, the elongation percentage at fracture is 6%, and the Young’s modulus is 73.5 GPa. More details can be found in ref. 37.

### Sample preparation

The manufacturing of specimens with natural defects consisted in the use of laboratory tomography to spot the presence of pores in the *ϕ* = 10 mm central region of 2.5 mm thick samples sliced out of the rods. Once a pore was detected, its position was marked and the fatigue specimens (with the gauge measuring 4 mm in height and a ~2 mm × 2 mm cross section) were carved out by Electrical Discharge Machining (EDM). Finally, the fatigue specimens were screened by performing laboratory *μ*CT scans, and the ones selected were ground (a layer ~300 *μ*m thick was systematically removed in order to avoid the presence of surface oxides created during EDM) and polished down to 0.02 *μ*m in order to ensure consistent surface finish with low residual stress. Laboratory tomography was carried out with a Phoenix Vtome tomograph where the source/detector distance is 577 mm. The voltage was 90 kV, the intensity 240 *μ*A (attenuation factor of 65%) and the voxel size = 4 *μ*m.

**Figure 7:**
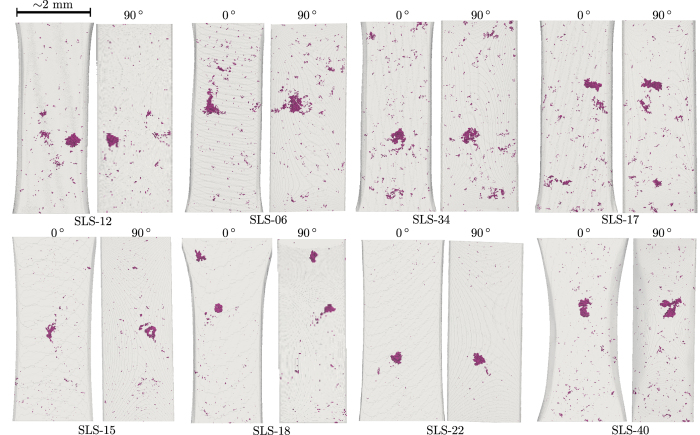
Several of the fatigue specimens produced for the *in situ* synchrotron experiments.

As shown in Fig. 7, specimen cross sections are small and so, the idea of *subsurface* pores is not straightforward (in other words, defects are never very far from the surface). In this study, a pore is considered as *surface* pore if there is contact with the ambient air. On the contrary, a pore is considered as *internal* when embedded within the bulk, whatever the distance to the free surface, so the pore is isolated from the ambient air. A specimen is considered suitable for fatigue testing when only internal pores are present within the gauge. Likewise, a specimen is ruled out when it contains both surface and internal pores, only surface pores or no pore at all (*i.e.,* when the pores contained within the gauge are smaller than 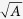 ≈ 100 *μ*m, as below this size pores are hardly observable using 4 *μ*m of voxel size). Overall, only 20–25% (in total, ≈100 fatigue specimens were screened) of the specimens produced following the manufacturing process described above were considered suitable. It must be kept in mind that this manufacturing process is material and time consuming. Further, in spite of having produced 20 suitable specimens (some of them are shown Fig. 7), only five of them could be tested because of the limited availability of beamtime at the synchrotron.

### *In situ* fatigue testing

The imaging of 3D microcracks requires synchrotron X ray *μ*CT. A 25 keV monochromatic X ray beam with 2% bandwidth is incident on the sample. The detector system is positioned 70 mm downstream the sample. The main contrast mechanism in this case is the near field diffraction of the partially coherent beam interacting with the sample. The X-ray photons are converted by a 100 *μ*m scintillator screen and guided into aCMOS detector via visible light objective lenses. The effective pixel size is 1.7 *μ*m which means that the isotropic voxel dimensions in the reconstructed tomographic images are of the same size. The results are 3D phase contrast enhanced attenuation maps. A dedicated fatigue machine is fixed onto the beamline rotation stage and sequential tensile testing (R = 0.1 at 10 Hz) is performed (more details about *in situ* testing can be found in ref. 35). Radiographic examination is carried out after each interval and prior to crack nucleation. Occasionally, *μ*CT scans are needed to endorse the radiographic examination. In spite of this, there are cases where the first stages of crack nucleation are overlooked. Once the crack nucleation detected, *μ*CT scans are regularly performed in order to record the crack progression. The number of cycles for every loading interval between scans is chosen depending on the remote stress level and the size of the propagating crack. Finally, each round of experiments (2 in total) consisted in 4 days of fatigue testing at the Swiss Light Source^41^.

## Additional Information

**How to cite this article:** Serrano-Munoz, I. *et al*. Location, location & size: defects close to surfaces dominate fatigue crack initiation. *Sci. Rep.* **7**, 45239; doi: 10.1038/srep45239 (2017).

**Publisher's note:** Springer Nature remains neutral with regard to jurisdictional claims in published maps and institutional affiliations.

## Figures and Tables

**Table 1:** Summary of all specimens tested during the synchrotron *in situ* fatigue testing.

Specimen Name	*σ*_*max*_ (MPa)	Internal Defect 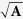 (*μ*m)	Cause of failure	N_*f*_ (cycles)
SLS-06	200	341 (no crack)	Surface defect (154 *μ*m)	82,000 (broken)
SLS-14	230	300 (crack at 40,000 cycles)	Surface defect (100 *μ*m)	73,000 (stopped)
SLS-17	200	386 (nucleation overlooked)	Surface defect (not within FOV)	190,000 (stopped)
SLS-22	200	314 (crack at 355,000)	Surface defect (not within FOV)	355,000 (broken)
SLS-40	240	350 (no crack)	Surface defect (80 *μ*m)	82,000 (stopped)

The Internal Defect 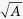 (*μ*m) value stands for the largest internal pore observed within specimens gauge. The size (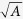) of some nucleating defects cannot be evaluated because they are located outside the Field of View (FOV).
